# Mental Health Reform: Design and Implementation of a System to Optimize Outcomes for Veterans and Their Families

**DOI:** 10.3390/ijerph191912681

**Published:** 2022-10-04

**Authors:** Andrea Phelps, Ellie Lawrence-Wood, Anne-Laure Couineau, Mark Hinton, Paul Dolan, Patrick Smith, MaryAnn Notarianni, David Forbes, Fardous Hosseiny

**Affiliations:** 1 Phoenix Australia—Centre for Posttraumatic Mental Health, Department of Psychiatry, University of Melbourne, Melbourne 3053, Australia; 2 Atlas Institute for Veterans and Families, Ottawa, ON K1Z 7K4, Canada

**Keywords:** mental health, veterans, service system

## Abstract

The social, health, and economic burden of mental health problems in the veteran community is heavy. Internationally, the array of services and support available to veterans and their families are extensive but vary in quality, are often disconnected, complex to navigate, and lack clear coordination. This paper describes a conceptual framework to guide the design and implementation of a system of services and supports to optimize the mental health and wellbeing of all veterans and their families. The framework recognizes the diversity of veterans across intersecting identities that uniquely shape experiences of posttraumatic mental health and wellbeing. It brings together several strands of research: the values and principles that should underpin the system; the needs of diverse veterans and their families; challenges in the current services and supports; evidence-based interventions; and principles of effective implementation. Central to the future system design is a next generation stepped model of care that organizes best and next practice interventions in a coherent system, matches service provision to level of need and addresses access and navigation. Practical guidance on implementation provides an aspirational and flexible structure for system evolution, and a template for all stakeholders—individuals, groups, agencies and organizations—to effect system change.

## 1. Introduction

In the context of military service where exposure to significant stressors or potentially traumatic events occurs frequently, posttraumatic mental health is an area of considerable importance. There are a range of normal physiological and psychological responses following stress and trauma exposure, including sleep disturbances, feelings of anger and guilt, psychological distress, social withdrawal, as well as more visceral physiological responses including hypervigilance to threat and emotional numbing. In the majority of individuals, these responses abate over time as the events are processed and integrated, and result in little functional impairment. However, in some individuals these symptoms can progress and worsen rapidly or gradually, resulting in the emergence of clinical disorders such as anxiety, depression and posttraumatic stress disorder (PTSD) [1,2,3]. There is substantial evidence of elevated rates of these posttraumatic mental health problems and disorders within veteran populations internationally [4]. 

The social, health, and economic burden of posttraumatic mental health problems in the veteran community is heavy. The social burden includes psychosocial stressors related to finance, housing and employment, family stress and breakdown, and social isolation [5,6,7]. The health burden arises from high mental health morbidity, associated physical health problems and high disability [6,7,8]. The economic burden is reflected in increasing compensation claims for mental health conditions, high healthcare costs and reduced productivity [9,10]. 

Currently, veterans and their families face an extensive array of services and supports, provided by government, not-for-profit, charity and veteran organizations. The services and supports available to veterans and their families differ across countries in terms of complexity, accessibility, cultural competency and capacity to provide specific evidence-based approaches. Few are organized and presented as a coherent system, leaving many veterans and family members unaware of services that would potentially best meet their needs. Further, in the absence of consistent quality assurance mechanisms, veterans and family members are at risk of receiving poor quality, poorly targeted and/or poorly coordinated support. There is an urgent need to optimize mental health and wellbeing outcomes for veterans and their families through an improved system. Therefore, the objective of the conceptual framework described in this paper is to provide a blueprint for future system design that addresses these challenges, and is supported by practical implementation goals. For the first time, the full range of services and supports available to veterans and their families, are conceptualized as an integrated system of care, based on a set of principles that should underpin services for veterans and their families, an understanding of their needs, recognition of challenges in the current system of supports, evidence-based interventions and the principles of effective implementation. The key elements of this system include taking a holistic wellbeing (rather than pathology-based) approach, integrating services for veterans with services for families, enabling direct access to the system at the appropriate level for current needs, incorporating digital resources alongside face-to-face care, and commitment to measurement-based care across the whole system.

The framework we have developed sets out a stepped/matched model of care that organizes best and emerging practice interventions. This model comprises services and supports in tiers of increasing intensity and specificity, with flexible entry and navigation through the system to best meet the needs of the individual and their family at a particular point in time. In considering not only the interventions required at each level of care, but also mechanisms that address entry into, navigation within and care-coordination across the system, it holds the potential for personalised care delivered flexibly to meet the ever-changing needs of veterans and their families. While this framework focusses on veteran mental health care, it is intended to be more broadly applicable, providing a framework for how to approach system reform in related areas, particularly where there are complex and competing demands and requirements, and an array of existing services and systems.

## 2. Principles Underpinning System Reform

The authors contend that the principles that should underpin and influence all elements of future system design for veterans and their families are: (1) respect and dignity, (2) engagement and involvement, (3) equity of access, (4) breadth of support, (5) high quality treatment and care, (6) holistic outcomes and (7) economic responsibility. Each of these complementary and overlapping principles are briefly described here. The principle of respect and dignity requires that each veteran and family member within the system is treated as an individual. This means acknowledging and respecting the heterogeneity of veterans and families in the etiology of mental health problems, their choices, goals, and priorities. The second principle, engagement and involvement, requires that the system promotes shared decision-making and the use of co-production/co-design models for services, with meaningful and authentic engagement of people with lived and living experience in all aspects of the system. To do this, it is necessary to establish reliable and bidirectional communication channels. The third principle, equity of access, requires addressing systemic inequities in provision, access and quality of care. This includes considering how aspects of identity, including but not limited to gender, race/ethnicity, socioeconomic status, occupational context, and geographical location, may intersect and influence participation and representation in systems and services. It also requires examining drivers of potential inequity at the level of system users, service providers, and within service system design and implementation. The fourth principle, breadth of support, requires the adoption of a broad approach to service delivery. This means going beyond narrowly focused models of mental health treatment and moving towards a more holistic approach to wellbeing, supporting veterans and family members in, for example, physical, social, and occupational domains. Such an approach is underpinned by a strong recovery focus, based on wellness, prevention, and early intervention. The fifth principle, high quality treatment and care, centres on the provision of evidence-based treatment options where available, and evidence-informed treatments where evidence gaps exist. Evidence gaps are addressed by building the evidence-base of what works, and explicitly acknowledging and addressing equity issues in the quality of treatments. Additionally, the system has a strong emphasis on pathways to care and care coordination. The sixth principle, holistic outcomes, requires that the system target a range of outcomes for veterans and family members, beyond symptom reduction, such as improvements in social and occupational domains, psychological wellbeing, and quality of life. The seventh principle, economically responsible, recognizes the obligation to make best use of scarce financial resources to achieve the best possible outcomes for veterans and family members as well as the broader economic and financial benefits that follow from a high performing system. This does not of course diminish the need for adequate funding for services and supports, a challenge in the mental health sector internationally [11]. 

## 3. The Needs of Diverse Veterans and their Families

Moving beyond these principles, an understanding of the needs of diverse veterans and their families is core to designing an ideal and high-functioning system of services and supports. Supporting the mental health and wellbeing of military populations has long been a priority for governments internationally, in recognition of the potential immediate and longer-term effects of military service on both veterans and their families. This includes the effects of military service across the life course, not only during active service, but also during and following transition out of service. 

Veterans and their family members have broad and varying needs in relation to mental health, shaped by their unique and intersecting identities as well as life experiences. An understanding of this diversity plays an important role in understanding their needs. The mental health needs of veterans relate to the whole spectrum of mental health disorders, subthreshold symptoms, as well as related problems including anger, aggression, guilt, social withdrawal, and emotional numbing [2]. Together these disorders, symptoms and related problems are associated with substantial impairment in social and occupational functioning and quality of life, and are likely to have reciprocal impacts across economic, social and other health domains [5,6,7,8,9,10]. Relative to veteran needs, less is known about the nature and prevalence of family needs. However, the impacts of military service extend beyond veterans themselves, with spouses or partners, children, relationships, and the family unit as a whole potentially affected [5]. Addressing the needs of family members is important not only in its own right but also because strong family functioning is supportive of veterans’ needs.

## 4. Challenges in Current Systems of Care for Veterans and their Families 

Internationally, there is consensus that existing systems of care for veterans do not adequately meet their needs across all areas [12,13,14]. While some elements of current systems are high quality, taken as a whole, they can often be complex, disjointed and difficult to navigate. Further, a range of challenges and barriers remain in areas of support, access to services and engagement in treatment. These include individual level factors such as personal attitudes and stigma, and more systemic barriers such as lack of access, inadequate treatment planning, lack of capacity, ineffective treatment, fragmentation and poorly coordinated care, gaps in evidence, and families and peers being not visible or leveraged [15,16,17]. A high-performing mental health system needs to be multi-component, multi-service, and interactive—spanning health, mental health and social care services—across the public, private and not-for-profit sectors.

## 5. Future System Design and a Model of Care

Based on the principles, needs of veterans and their families, and challenges in the current system of care, there are several features that should be considered in the design of the future system. First amongst these and the central organizing principle is that the veteran and their family should stand at the centre of the system. This means that support should be provided as close to home as possible, services are culturally competent, and veterans and families have a say in service design and delivery. It also means that services and supports are both accessible and acceptable to the diverse veteran community, with help available across a range of service delivery modes, including digital channels, and tailored to individual need. Multiple strategies should be used to make access easier for veterans and their families seeking assistance. Peers have a critical role to play, for example, in steering someone towards services. In terms of access, there should be a culture of inclusion with a low threshold for entry. This is important for successful early identification of distress and impairment and for encouraging veterans to seek treatment. 

Upon entry, the system should have the capacity to provide high quality and timely assessment and treatment planning. This requires competent clinicians with a thorough understanding of military life/culture and associated mental, physical and psychosocial issues, as well as a thorough knowledge of the full range of available services. System navigation, as distinct from care coordination, should be an acknowledged function and skill set. The system also requires networks of treatment excellence with a range of treatment options and modalities, ideally accessible via a single point of entry. 

Following assessment and treatment planning, high quality treatment should be the cornerstone of care. Evidence-based treatment options and measurement-based care should be embedded across the system and outcome data used to inform which services should continue or be scaled-up. Although a range of individuals and agencies will provide services, they need to be mapped and well-coordinated in order to provide a seamless pathway for veterans and their families moving between different parts of the system including across mental and physical health. This requires the best possible care coordination models that facilitate communication between providers, service navigators, veterans and their families. 

Big data and advanced analytics techniques, including AI and machine learning, should be employed to drive evidenced-based decision making. In addition, clinical research should guide continuous improvement, with a knowledge translation program in place to reduce the time taken to embed innovative treatments into clinical practice. 

Key system levers around regulation, funding, policy, programs, workforce reform and performance should be aligned and working towards the collective goal. This means that funding models across the wellness and care continuum should incentivize access and effective treatment of veterans and their families. There should be consideration of outcomes-based funding, accompanied by adequate funding for workforce capability building in measuring outcomes, and leadership support. Funding for research to address gaps in evidence and guide future ‘next’ practice, as well as translational research and practice-research partnerships, would further support improved outcomes for veterans and their families. 

Long-term financial sustainability and value-based care across the system needs to be a focus, balancing efficiency with desired outcomes, experience and service quality. Accordingly, there should be consistent standards and performance metrics across service providers, for various quality indicators such as access, quality/safety, outcomes, and finances.

Improved outcomes for veterans and their families, requires all stakeholders to be involved in system co-design, implementation and operation. This includes veterans and families with lived experience of posttraumatic mental health issues and treatment, the broader veteran community, peer supporters, and health and wellbeing professionals. System design and operation also requires close collaboration with, agencies providing adjacent services in other areas including physical health, occupational rehabilitation and support, housing, and financial counselling. Finally, the importance of the funding and policy context of the service system needs to be acknowledged, with the involvement of key government decision makers and funding bodies in service system reform. 

Fundamental to the success of the system is the provision of best and next practice interventions. A stepped/matched model of care that helps to organize these interventions is described in the following section.

## 6. Stepped/Matched Model of Care

**Figure 1:**
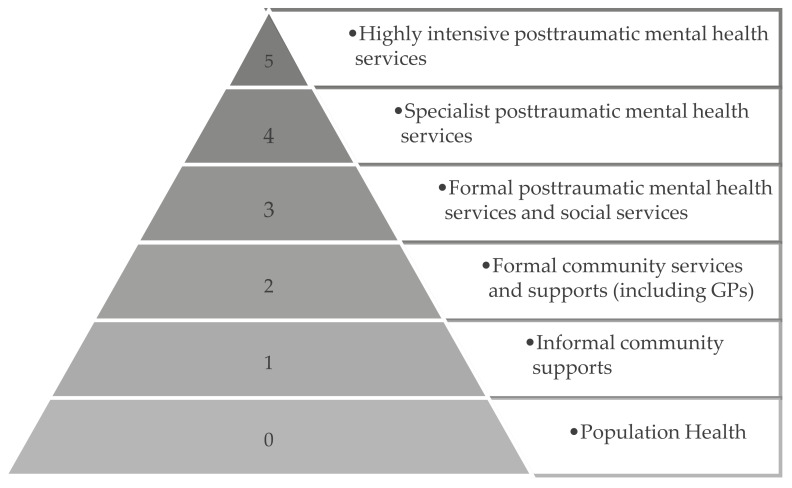
Overview of Tiers.

The stepped/matched model of care is comprised of multiple components (tiers) operating both within and outside traditional mental health settings (See Figure 1). While some veterans might only access one tier of the model—a brief episode of treatment, for example—many will access multiple services across several tiers. Each tier contains several types of interventions, and the level of evidence for interventions varies across tiers, with a trade-off often apparent between reach and effectiveness [18] (see Supplementary Table S1. using interventions for PTSD as an example).

At the lower tiers, interventions target the whole population (universal interventions) and are intended to have broad reach. These may include, for example, online psychoeducation materials and self-help digital resources. At a population level, improved outcomes can be achieved if a large number of veterans and families access such resources, even if they are only minimally effective. At the higher tiers, evidence-based treatments delivered by specialized practitioners reach fewer veterans but are more effective. Both reach and effectiveness are important ways of measuring outcomes for veterans and family members at a population level. The proposed stepped/matched model creates a dynamic system that optimises outcomes at a population level, by focusing equally on reach, uptake, engagement and outcomes [18].

At the higher tiers, evidence-based practice (EBP) is core. EBP strives to integrate the best research and evidence with clinician expertise, as well as patient preferences and values [17] to achieve higher quality care, improved patient outcomes, reduced costs, and greater staff and patient satisfaction. Across all tiers, EBP involves engaging staff and service users in identifying practices that could be improved, identifying barriers and enablers, designing and implementing interventions based on research evidence and outcome data, reviewing and adjusting the intervention as required, and designing strategies to maintain change [17].

### 6.1. Key Features of the Stepped/Matched Model

The success of the model relies on several important features. The first feature is comprehensive multimodal individual and family assessment at the point of entry. This assessment aims to match veterans and family members to optimal services and supports that will best meet their needs. It also encourages shared decision-making in treatment planning and is crucial to the engagement of veterans and family members in the system. The second feature is the availability of acute assessment and intervention to all veterans and their families with sudden exacerbations in need, regardless of whether they are already closely linked with a specialist service. The outcome of this assessment should be timely access to the level of care indicated. The third feature is system navigation. A stepped/matched model of care assumes a system of care that includes multiple components across different settings, and at differing levels of intensity. System navigators should be familiar with all components of the system, but also know the veteran and family well. These navigators have a role in assisting the veteran and family to access the mental health system as well as the many other departments and agencies with which they will need to engage [19]. The fourth feature is care coordination, which recognizes the challenges inherent in a system with multiple points of entry into and pathways through various services and supports. The fifth feature is a holistic view of the system and integrated pathways, which recognizes that it needs to be explicitly linked in with broader wellbeing services and supports within the community. The sixth feature is priority given to research, evaluation, and continuous improvement across all levels of the system. A detailed schematic representation of the system is available in Supplementary Figure S1.

### 6.2. Considerations for Implementing the Model

A number of implementation challenges identified in the literature have been considered in the development of an effective and practical approach to implementation of the stepped/matched model of care. First, effecting change in complex systems is difficult and the delivery of effective treatments within health systems has been identified as one of the most difficult challenges [20]. It requires a holistic approach that involves consumer engagement, research and evaluation design, policy development and clinical governance and practice [21]. Second, the traditional research approach that progresses through clinic-based efficacy research to real-world effectiveness trials and then dissemination is problematic [15,16]. It leads to long delays in the adoption of effective programs [22] and is often not tailored to local needs and practices. [23]. Translation experts have called for new approaches to integrating research with practice include action research, participatory research models, and implementation/evaluation research [15,16,24]. Third, effective implementation requires cross-sector change and collaboration across multiple leadership structures [16].

Knowledge mobilization and implementation science are two closely related fields that aim to address these challenges through an iterative cycle between research and practice—new knowledge informs practice, and practice informs what knowledge is produced and how it is best applied.

Drawing on the knowledge mobilization and implementation science literature, we have identified six building blocks for effective implementation of the system of care. Firstly, leadership is of central importance to effective implementation [25]. With multiple leadership structures involved in developing and maintaining an evidence-based system of care for veterans and their families, there needs to be collaboration between leaders if implementation is to be effective [16]. Intermediary organizations can play a role in bringing leaders together to work collaboratively on implementation, and to contribute to and draw upon their pooled influence. Intermediary organizations may include organizations that have tested and packaged EBPs (purveyors), research institutes that focus on translation, non-governmental service providers or government agencies that deliver services or act as regulators [26]. These organizations often act as a bridge between researchers, government decision makers, clinical leaders and the community. They can also help build leaders’ implementation capability through brokerage services, leadership training and implementation planning tools [27].

Secondly, collaboration between researchers, practitioners and veterans needs to be maximised. A disconnect between knowledge creation (research) and use (practice) can result in the development of interventions without consideration for the context in which they will be delivered [16]. Implementation is more likely to be effective when practitioners and clients are engaged in research from the outset [24]. Similarly, active collaboration with service-users in the planning and review of services is also important in the implementation of new practices [28]. 

Thirdly, implementation planning should address access to care for veterans who experience health care barriers [29]. This can be achieved by ensuring marginalised community members are included in stakeholder engagement and that the voices of diverse veterans and their families are included in all aspects of research, program design and roll out [28]. Furthermore, there should be data-driven understanding of the needs of disadvantaged veterans, that allows government decision makers, policy actors, researchers and service providers to set priorities informed by health inequities [28]. 

Fourthly, there is a need to build capacity and capability of all elements of the system (providers, organizations, and peer supporters) to conduct assessments, make informed decisions about care planning, and deliver evidence-based treatments, consistent with their role. Towards this end, the stepped/matched model of care can be used as a tool for each player in the system to consider the services and supports that they offer and to map those alongside the services and supports offered by others.

From a provider perspective, capacity development involves fostering both the knowledge and skills to deliver services as intended, and attitudes that are core to the service delivery model, e.g., client-centred values [30]. For example, attitudes about self-efficacy, change, and evidence-based practice have been targeted through implementation training and capacity development [31,32]. Looking beyond the provider perspective to the context of large scale implementation, capacity-building programs must consider the knowledge and skill needs of practitioners in the context of organizational resources, climate and culture [24]. Capacity building interventions should not rely on training alone but aim to integrate learning into day-to-day practice, using strategies such as ongoing monitoring of practice. One promising model of integrating learning into practice is the learning collaborative, where a network of practitioners is supported to continuously monitor the quality of their work as they implement a new practice [33].

Fifthly, an effective implementation process has to be able to be adapted to different systems, policy and funding environments and adaptations to EBPs made by service providers over time [23]. This can be achieved through a continuous quality improvement approach in which organizations use data to monitor outcomes and measure the impact of adaptations. Ongoing assessment of the context in which recommended practices are being embedded is included in most process implementation models (e.g., EPIS [34] or PRISM) and recognized implementation strategies (e.g., ERIC’s expert generated list of strategies) [35]. An effective form of outcome monitoring and feedback process is measurement-based care (MBC), which involves using clinical outcome and process data to inform clinical decision making. MBC has been found to improve clinical outcomes [36] and treatment fidelity [37], and supports shared decision making with service users and implementation of evidence-based practice. Building a culture of quality assurance and improvement across all levels of the system through communication and promotion of the benefits of MBC is key to support and sustain improved practice and implementation of EBPs. An intermediary organization is ideally placed for helping services adapt to change by facilitating implementation planning, supporting data collection and providing guidance on continuous quality improvement.

Finally, the use of data in an ongoing way to monitor and adjust implementation efforts is important for successful long-term implementation of EBPs [32]. Data about client outcomes, the implementation process, service delivery and utilisation should: (1) inform service planning and implementation requirements over time; (2) monitor and provide feedback on quality of care; and (3) measure implementation outcomes including the impact of different strategies. Implementation studies describe numerous feedback mechanisms, including meetings among stakeholders to discuss issues such as implementation barriers and facilitators and emerging concerns, and direct individual feedback to practitioners. Where individual feedback is used to embed new practice, regular data reporting can be used to highlight where implementation goals have and have not been met, which leads to working collaboratively with the practitioner to identify local barriers and mitigation strategies [38]. This iterative assessment process aligns with quality assurance processes [24,39].

## 7. Limitations and Future Directions

The framework outlined in this paper has been informed by the Australian and Canadian military contexts, and is intended to be broadly applicable across Western developed country contexts. Therefore, how it might translate to, and be applied in other cultural contexts is not known and an important consideration for future research. Furthermore, this framework is conceptual in nature, and its application in practice is currently untested. The framework clearly outlines a structure around which current and future initiatives in service development can be considered, and a template for identifying where services and systems across a range of different contexts currently sit in relation to best and next practice services and support. It also provides a guide to the most appropriate system architecture (system level); culture, workforce, systems and processes (organisational level); and behaviours and competencies required at an individual practitioner level to support implementation. In the future, it will be critical to pilot this framework within the complexity of existing service systems.

## 8. Conclusions

Our obligation to serve those who have served their countries makes the mental health and wellbeing of veterans and their families an issue of highest priority. Although there are many services and supports in existence, they are often limited in their availability, cultural awareness, and coordination, not presented as a coherent system with ease of access and navigation, and not subject to consistent quality assurance mechanisms. We have argued that there is a need for a future system whose design and development is underpinned by a set of core principles, and guided by several strands of research that have not previously been brought together in this way. This includes the identified needs of veterans and their families, shifting the emphasis from symptom reduction towards a more holistic approach of wellbeing, recognition of current challenges in service and supports, integration of evidence-based care and adoption of best practice principles for implementation. The authors contend that this is what is required to optimize outcomes for our veterans and their families, and for significant long-term savings for the system. 

## Data Availability

This paper does not report data.

## Supplementary Materials

The following are available online at https://www.mdpi.com/article/10.3390/ijerph191912681/s1, Figure S1: A Veteran-centric high-performing posttraumatic mental health system, Table S1: Example PTSD EBP menu organised by Tiers and levels of evidence.
