# Supplementary material for: Mental Health Reform: Design and Implementation of a System to Optimize Outcomes for Veterans and Their Families
Source: Int J Environ Res Public Health. 2022 Oct 4;19(19):12681. doi: 10.3390/ijerph191912681 (PMC9565186; doi:10.3390/ijerph191912681)
Supplement: Supplementary file 1 [file ijerph-19-12681-s001.zip › ijerph-1885972-supplementary.pdf]

Table S1. Example PTSD EBP menu organised by Tiers and levels of evidence

	**Tier 0** Population Health	**Tier 1** Informal community supports	**Tier 2** Formal community services and supports (including GPs)	**Tier 3** Formal posttraumatic mental health services and social services	**Tier 4** Specialist posttraumatic mental health services	**Tier 5** Highly intensive posttraumatic mental health services
**Interventions with high evidence of impact, that we can be confident of as first line best practice.**		▪ Social connectedness programs		▪ Cognitive behaviour therapy (CBT)<br>▪ Cognitive processing therapy (CPT)<br>▪ Cognitive therapy (CT)<br>▪ Eye movement desensitization and reprocessing (EMDR)<br>▪ Prolonged exposure (PE)<br>▪ Trauma-focused CBT (TF-CBT)	▪ Cognitive processing therapy (CPT)<br>▪ Cognitive therapy (CT)<br>▪ Eye movement desensitization and reprocessing (EMDR)<br>▪ Prolonged exposure (PE)<br>▪ Trauma-focused CBT (TF-CBT)<br>▪ Collaborative chronic care models (CCMs)	
**Interventions with moderate evidence of impact, which may require some further research and evidence to confirm this.**	▪ Mass media public health campaigns<br>▪ Public anti-stigma campaigns	▪ Reducing Stigma program - R2MR<br>▪ Reducing Stigma program - TWM<br>▪ Reducing Stigma program - Opening Minds	▪ Low intensity CBT<br>▪ On-line therapies<br>▪ Self-help digital applications<br>▪ Bibliotherapy<br>▪ Telephone support services<br>▪ Pharmacology – short term<br>▪ Single session or brief A&D treatments<br>▪ Community and peer led support groups<br>▪ Low intensity peer-support (peers trained)	▪ Psycho-education<br>▪ Brief therapy	▪ Narrative exposure therapy (NET)<br>▪ Present-centred therapy (PCT)<br>▪ Stress inoculation training (SIT)<br>▪ Group TF-CBT<br>▪ Guided internet-based trauma-focused CBT<br>▪ Selective serotonin reuptake inhibitors (SSRIs)<br>▪ Venlafaxine	▪ Trauma Recovery Programs (Aus)<br>▪ Intensive Treatment Programs (UK)
**Interventions with low evidence of impact require further research to determine with confidence their true impact.**	▪ Targeted Military mental health public awareness campaigns				▪ Couples trauma-focused CBT<br>▪ Group and individual (combined) TF-CBT<br>▪ Single-session trauma-focused CBT<br>▪ Non-trauma-focused CBT<br>▪ Meta-cognitive therapy<br>▪ Reconsolidation of traumatic memories (RTM)<br>▪ Virtual reality therapy (VRT)<br>▪ Written exposure therapy (WET)<br>▪ Ketamine, Quetiapine<br>▪ Repetitive transcranial magnetic stimulation (rTMS)<br>▪ Neurofeedback<br>▪ Mindfulness-based stress reduction (MBSR)<br>▪ Transcendental Meditation (TM)<br>▪ Acupuncture<br>▪ Yoga, Physical exercise	

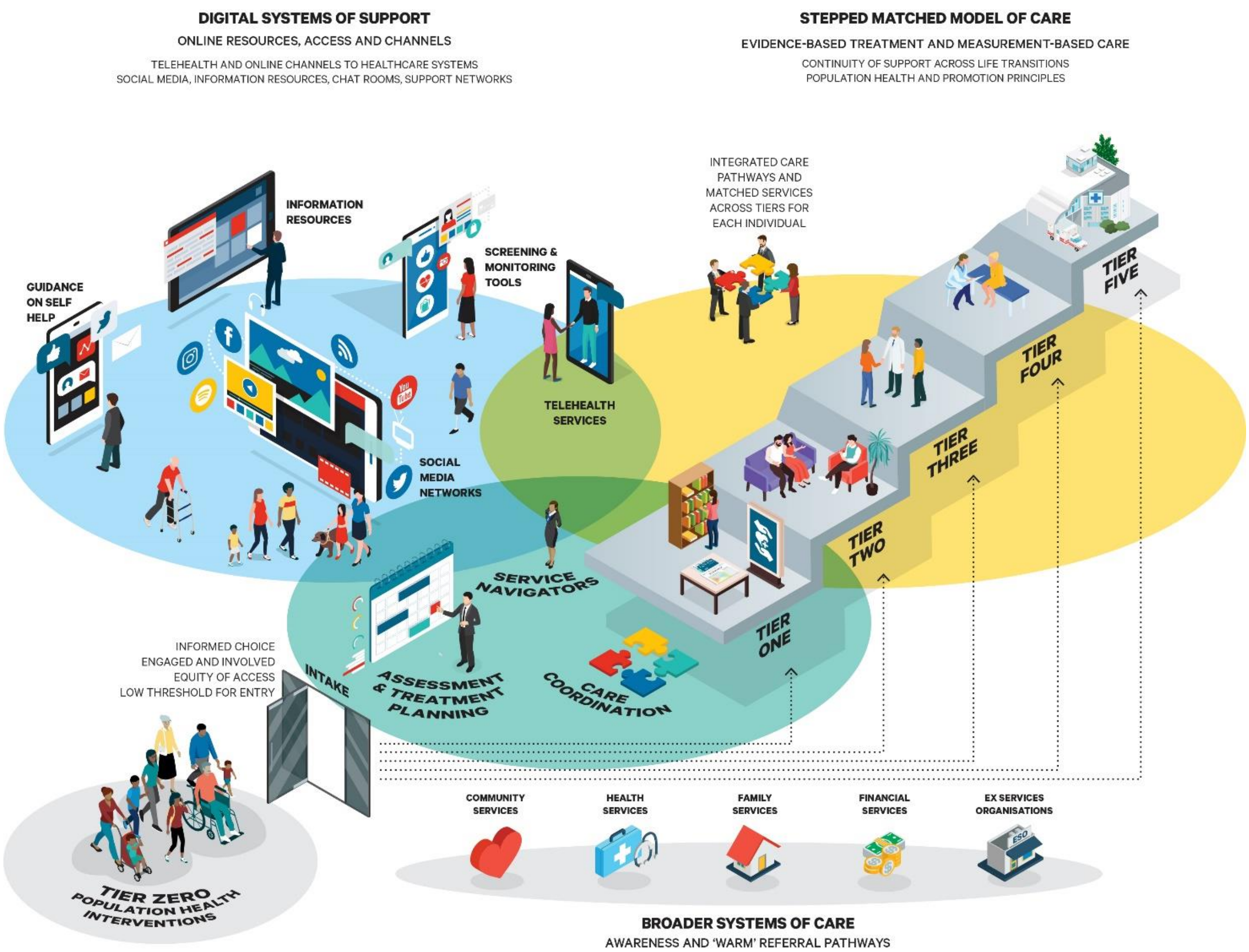

Figure S1. A Veteran-centric high-performing posttraumatic mental health system.
